# Global, regional, and national burden of atrial fibrillation and its association with alcohol consumption: age, sex, regional, and SDI differences—an analysis of the Global Burden of Disease Study 2021

**DOI:** 10.3389/fcvm.2026.1583980

**Published:** 2026-07-29

**Authors:** Ping Lai, Jinhua Ye, De-Kuan Zhang, Jia-Yuan Ling, Ke-Jun Tian, Bei Wang, Hong-Zhou Zhang, Yongling Liao, Jinhua Xue

**Affiliations:** 1 Department of Cardiology, First Affiliated Hospital of Gannan Medical University, Gannan Medical University, Ganzhou, Jiangxi, China; 2 Key Laboratory of Prevention and Treatment of Cardiovascular and Cerebrovascular Diseases, Ministry of Education, Gannan Medical University, Ganzhou, China; 3 Department of Pathophysiology, School of Basic Medicine, Gannan Medical University, Ganzhou, China; 4 The First School of Clinical Medicine, Gannan Medical University, Ganzhou, China

**Keywords:** alcohol consumption impact, atrial fibrillation mortality, global health trends, regional disparities in cardiovascular disease, socio-demographic index

## Abstract

**Background:**

Atrial fibrillation (AF) is a leading cause of mortality and disability globally. While trends in AF-related deaths are well-documented, the influence of alcohol consumption and regional disparities, particularly across different Socio-Demographic Index (SDI) regions, remains underexplored.

**Objective:**

To analyze global and regional trends in AF-related mortality from 1990 to 2021, focusing on age, sex, and the impact of alcohol consumption across regions and SDI.

**Methods:**

Data from the Global Burden of Disease (GBD) Study 2021 were used to assess age- and sex-specific AF mortality rates in 204 countries. Mortality trends were analyzed from 1990 to 2021, with attention to age group (30–44, 45–64, 65–84, 85–94, 95 + years) and sex. The impact of alcohol on AF-related mortality was examined by WHO regional classifications.

**Results:**

AF-related mortality increased globally in both men and women, with a higher rate in women. Female mortality increased notably in the 90–94 and 95 + age groups, while male mortality surged in the 30–44 and 85+ age groups. Regionally, Greenland showed a decline in male mortality, while Sweden's rates rose. In China, both male and female mortality rates decreased. Alcohol consumption had a significant impact on AF mortality in Europe and the Americas, with smaller effects in Africa, Southeast Asia, and the Eastern Mediterranean.

**Conclusion:**

The global burden of AF-related mortality is rising, with significant age, sex, and regional disparities. Alcohol consumption contributes notably to AF deaths in Europe and the Americas, highlighting the need for targeted interventions in high-risk regions. These findings emphasize the importance of addressing alcohol-related AF mortality through region-specific public health strategies.

## Introduction

Atrial fibrillation (AF) is the most common sustained arrhythmia worldwide, with a rising prevalence due to the aging population and the increasing burden of cardiovascular risk factors (1). The Global Burden of Disease (GBD) study has consistently shown that AF contributes significantly to morbidity and mortality, particularly through its association with stroke, heart failure, and cardiovascular death (2). Recent studies highlight that AF-related mortality has been rising globally, although the rates vary significantly by region, sex, and age (3). Understanding the spatiotemporal trends in AF mortality is crucial for identifying high-risk populations and informing targeted public health strategies.

In 2021, the GBD study expanded its scope to provide more granular data on the global burden of AF, enabling a detailed analysis of regional, sex-, and age-specific mortality trends (2). These trends reveal that while AF mortality has increased in many high-income regions, low- and middle-income countries (LMICs) are experiencing a more rapid rise in AF-related deaths. This rise is likely attributed to improved life expectancy and the increasing prevalence of risk factors such as hypertension, diabetes, and obesity (4). Furthermore, AF-related mortality is influenced not only by traditional cardiovascular risk factors but also by social determinants of health, such as alcohol consumption, which has been shown to both trigger and worsen AF.

Alcohol consumption, particularly in high-income countries, remains a major modifiable risk factor for AF (5). Alcohol has been linked to AF through multiple mechanisms, including the promotion of electrical disturbances in the heart, changes in atrial structure, and increased sympathetic nervous system activity (6). The global impact of alcohol on AF-related mortality varies by region, with European and Western Pacific regions showing particularly high mortality rates associated with alcohol use (5, 7). In contrast, regions with lower Socio-Demographic Index (SDI) scores, such as Africa and Southeast Asia, exhibit relatively stable and lower mortality rates from AF, though these regions still face growing health challenges as economic development progresses.

Despite recent advances in understanding AF mortality, several gaps remain in our knowledge of the broader social and environmental factors that influence AF outcomes across different populations. This study aims to bridge these gaps by analyzing the global, regional, and age- and sex-specific trends in AF mortality over the past three decades using the latest 2021 GBD data. Additionally, we explore the impact of alcohol consumption on AF-related deaths in varying SDI regions, providing novel insights into how socio-economic and lifestyle factors interact with clinical risk factors to shape the global burden of AF. Understanding the role of alcohol consumption in AF-related deaths is crucial for developing targeted public health interventions to mitigate the rising burden of AF, especially in high-risk regions.

## Methods

### Study design and data sources

This study is a retrospective, population-level analysis of AF-related mortality using data from the Global Burden of Disease (GBD) Study 2021. The GBD study is a comprehensive and systematic assessment of global health trends, covering a wide range of diseases and injuries, including AF, across all countries and territories. The GBD data are publicly available and provide age-, sex-, and region-specific mortality rates, as well as associated disability-adjusted life years (DALYs) for 204 countries and territories from 1990 to 2021.

We focused specifically on AF-related deaths, stratified by age, sex, and region, in order to assess global, regional, and temporal trends. The analysis incorporated data from multiple sources, including national vital registration systems, surveys, and health reporting institutions, ensuring a broad representation of mortality data across high-income, middle-income, and low-income countries.

### Inclusion and exclusion criteria

The study included all deaths attributed to AF, as defined by the International Classification of Diseases (ICD) codes I48.0–I48.9, which cover both primary and secondary AF, including complications such as stroke and heart failure. We excluded deaths that were not directly attributable to AF or were coded under different classifications (e.g., those related to other types of arrhythmias or cardiovascular diseases).

### Mortality estimation

AF-related mortality rates were estimated using a combination of the cause of death methodology and disability-adjusted life years (DALYs). The GBD study estimates cause-specific mortality by utilizing a Cause of Death Ensemble model (CODEm), which integrates multiple data sources to generate consistent and reliable estimates. The model incorporates data from national vital registration, verbal autopsy, and other mortality surveillance systems. In addition to death counts, the GBD team estimates DALYs for AF, which accounts for both premature mortality and years lived with disability (YLDs) due to AF-related complications.

### Age and sex stratification

Mortality data were stratified by age group (30–44, 45–64, 65–84, 85–94, 95+years) and sex (male and female), in accordance with the GBD's established age categories. Age-specific mortality rates were calculated for each sex and region, and temporal trends from 1990 to 2021 were analyzed.

### Regional stratification

Regional stratification followed the SDI classification, which categorizes countries into five regions based on their level of social and economic development. The SDI incorporates income, educational attainment, and fertility rates to classify regions as having low, low-middle, middle, high-middle, or high SDI. AF-related mortality trends were compared across regions and SDI categories to explore the impact of socioeconomic development on AF outcomes.

### Alcohol consumption analysis

We analyzed the influence of alcohol consumption on AF-related mortality using WHO regional classifications. Alcohol-related mortality rates for AF were obtained from the GBD study's alcohol-attributable fraction estimates, which are based on regional drinking patterns, prevalence of alcohol use disorders, and its relationship to AF and other cardiovascular diseases. The regions examined included Europe, the Americas, the Western Pacific, Southeast Asia, Africa, and the Eastern Mediterranean. This analysis was conducted to evaluate regional differences in alcohol-related AF mortality and to examine potential regional trends.

### Statistical methods

All analyses were conducted using publicly available GBD data and standardized statistical methods. The mortality rates were expressed per 100,000 population. Temporal trends were evaluated using linear regression to assess the annual rate of change in AF-related mortality between 1990 and 2021. Statistical significance was assessed using a *p*-value of <0.05. Additionally, age-standardized mortality rates were calculated to account for population age structure differences across regions.

For regional and sex-specific analyses, we applied age-standardization methods to control for variations in population age distribution. The impact of alcohol consumption on AF mortality was evaluated using relative risk estimates for alcohol-attributable deaths, adjusted for confounding factors such as comorbidities (e.g., hypertension, diabetes) and healthcare access.

### Sensitivity and robustness checks

To assess the robustness of our findings, we performed sensitivity analyses using alternative models of cause attribution and mortality estimation. We also conducted checks for missing data and outliers, ensuring that the estimates were consistent with reported trends in AF mortality across different datasets (2).

### Ethical considerations

This study utilized publicly available aggregated data from the Global Burden of Disease Study, and therefore, no direct patient-level data were accessed. As such, ethical approval was not required for this study. All data used in this study were de-identified and anonymized in compliance with ethical standards for global health research.

## Results

### Age group distribution and regional differences in AF related global mortality

**Figure 1:**
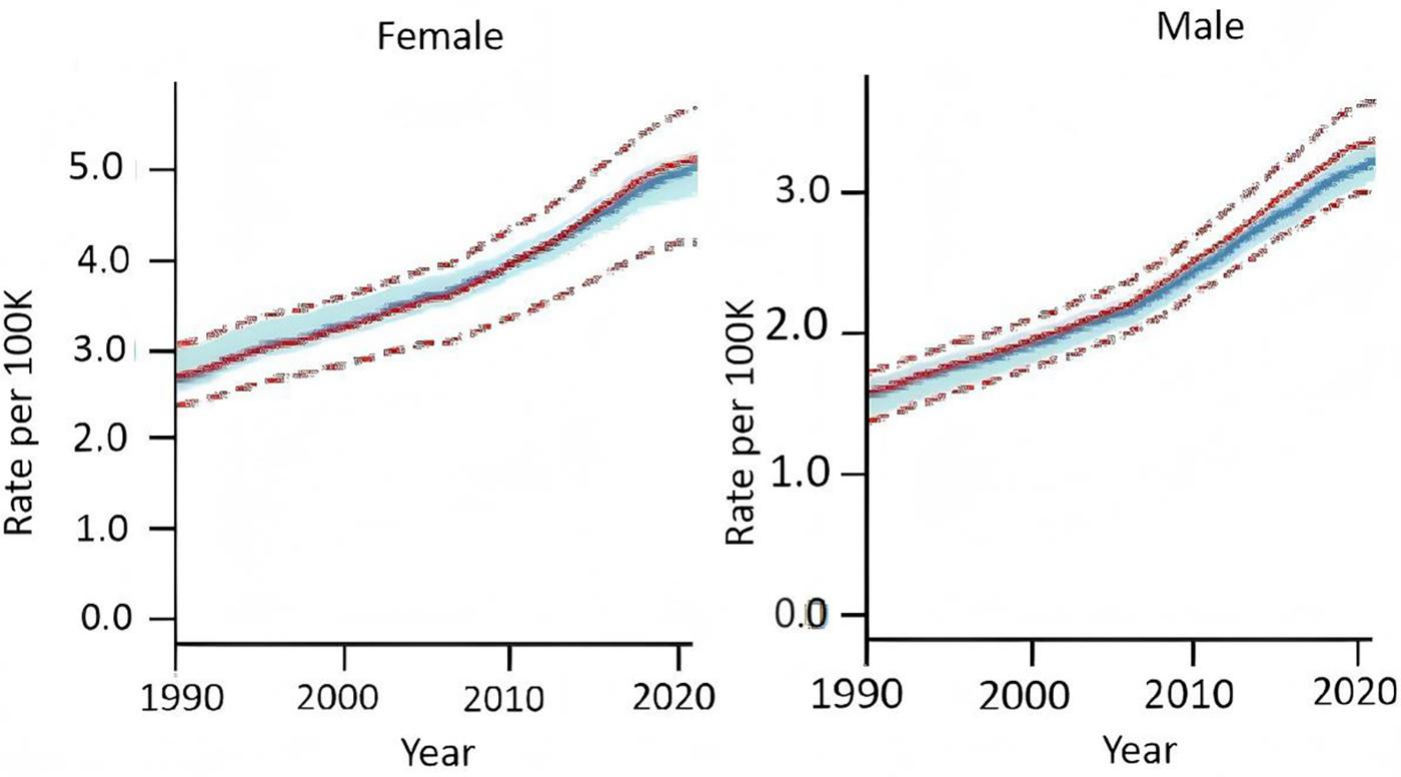
Global trends in age-standardized atrial fibrillation (AF)-related mortality by sex, 1990–2021. Data source: Global Burden of Disease (GBD) Study 2021. Case definition: AF-related deaths coded under ICD-10 I48.0–I48.9, encompassing primary and secondary AF as well as complications including stroke and heart failure. Metric: Age-standardized mortality rate per 100,000 population, calculated using the GBD standard reference population. Model: Mortality estimates were derived from the Cause of Death Ensemble model (CODEm), which integrates national vital registration systems, verbal autopsy, and mortality surveillance data. Adjustment: Estimates are standardized for age and sex; comorbidities (e.g., hypertension, diabetes) and healthcare access were incorporated as covariates within the GBD modeling framework. Uncertainty: Shaded areas represent 95% uncertainty intervals (UI).

**Figure 2:**
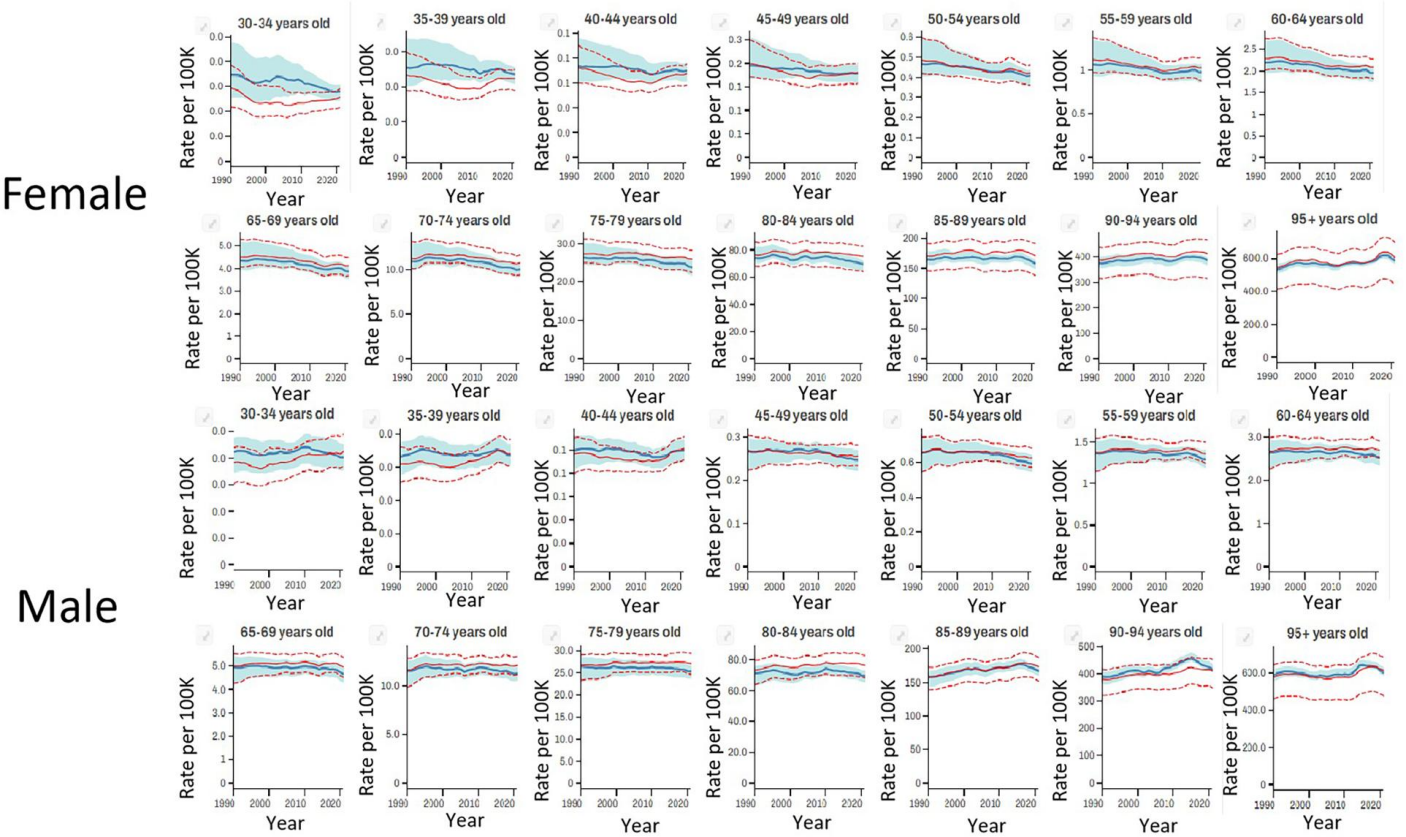
Age- and sex-specific trends in global AF-related mortality, 1990–2021. Data source: GBD Study 2021. Case definition: ICD-10 I48.0–I48.9 (primary and secondary AF, including stroke and heart failure complications). Metric: Age-specific mortality rate per 100,000 population. Age groups: 30–44, 45–64, 65–84, 85–94, and 95+ years. Model: CODEm, integrating multiple mortality data sources with covariate-based ensemble estimation. Adjustment: Age- and sex-specific estimates; comorbidities and healthcare access adjusted within the GBD framework. Uncertainty: Shaded ribbons denote 95% UI. Key observations: Female mortality increased notably in the 90–94 and 95+ age groups, whereas male mortality surged in the 30–44 and 85+ age groups.

The global mortality due to AF has been increasing in both men and women, with a similar rate of increase in both genders. However, the mortality rate in women is slightly higher than in men (Figure 1). Specifically, in women, the increase in mortality is mainly observed in the 90–94 years and 95+ years age groups, while the mortality in the 80–89 years group remains stable, and the 30–79 years group shows a decreasing trend. In men, the mortality rate rises in the 30–44 years and 85+ years groups, while the 45–64 years group shows a slight decrease, and the 65–84 years group remains stable (Figure 2).

**Figure 3:**
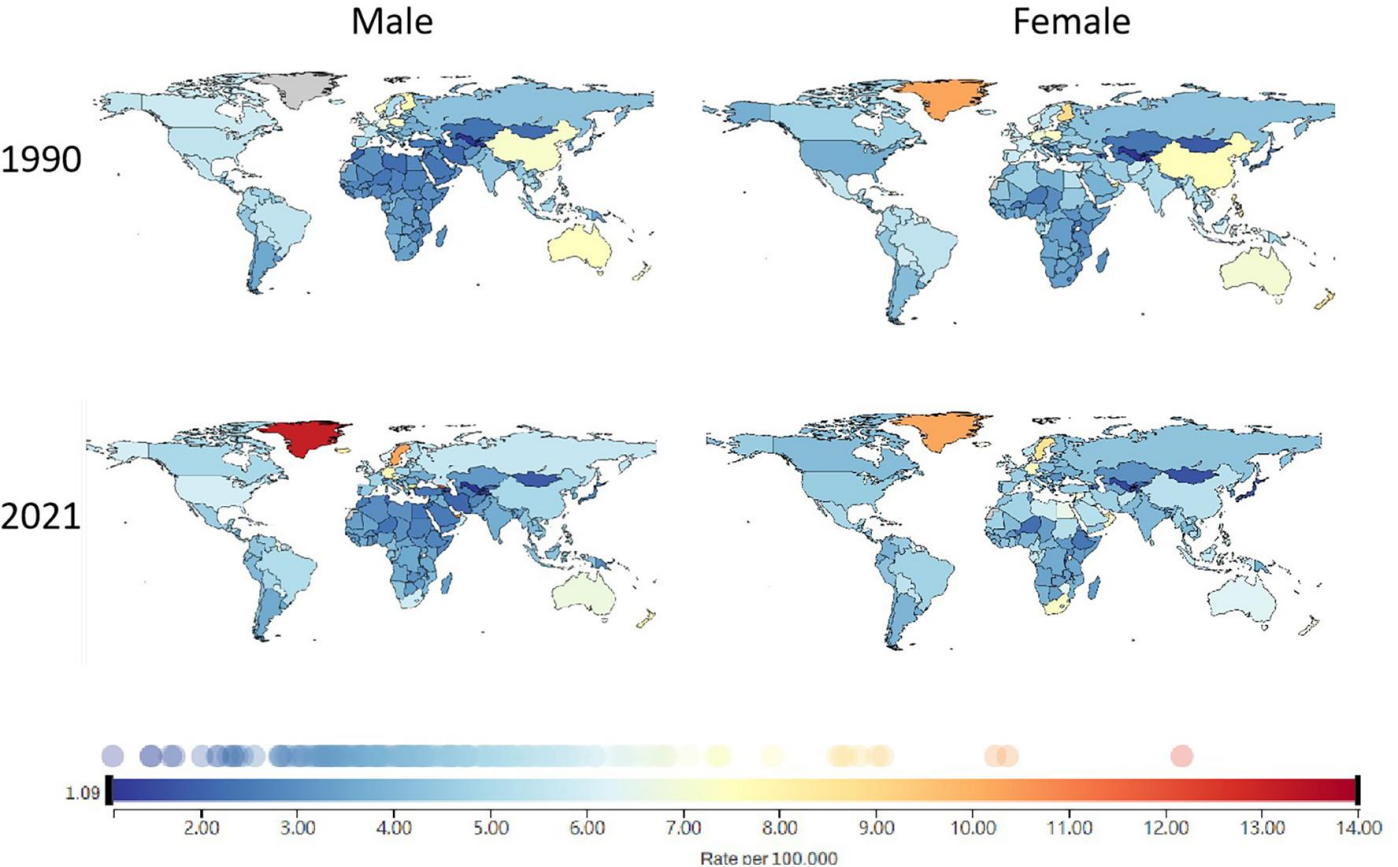
National trends in age-standardized AF-related mortality for selected countries and territories, 1990–2021. Data source: GBD Study 2021. Case definition: ICD-10 I48.0–I48.9. Metric: Age-standardized mortality rate per 100,000 population. Model: CODEm ensemble estimates. Adjustment: Age- and sex-standardized; comorbidities and healthcare access covariates included in GBD models. Uncertainty: Error bands represent 95% UI. Illustrative examples: Greenland (male mortality declined from 15.646 to 13.238 per 100,000), Sweden (male mortality increased from 5.054 to 10.436 per 100,000), and China (male mortality decreased from 7.251 to 5.220 per 100,000; female mortality decreased from 6.874 to 5.325 per 100,000).

The mortality rate due to AF has changed between 1990 and 2021 in different regions. For instance, the male mortality rate in Greenland decreased from 15.646/100,000 to 13.238/100,000, while in Sweden, it increased from 5.054/100,000 to 10.436/100,000. In China, the male mortality rate decreased from 7.251/100,000 to 5.220/100,000, and the female mortality rate decreased from 6.874/100,000 to 5.325/100,000 (Figure 3).

### Impact of alcohol in AF related mortality

**Figure 4:**
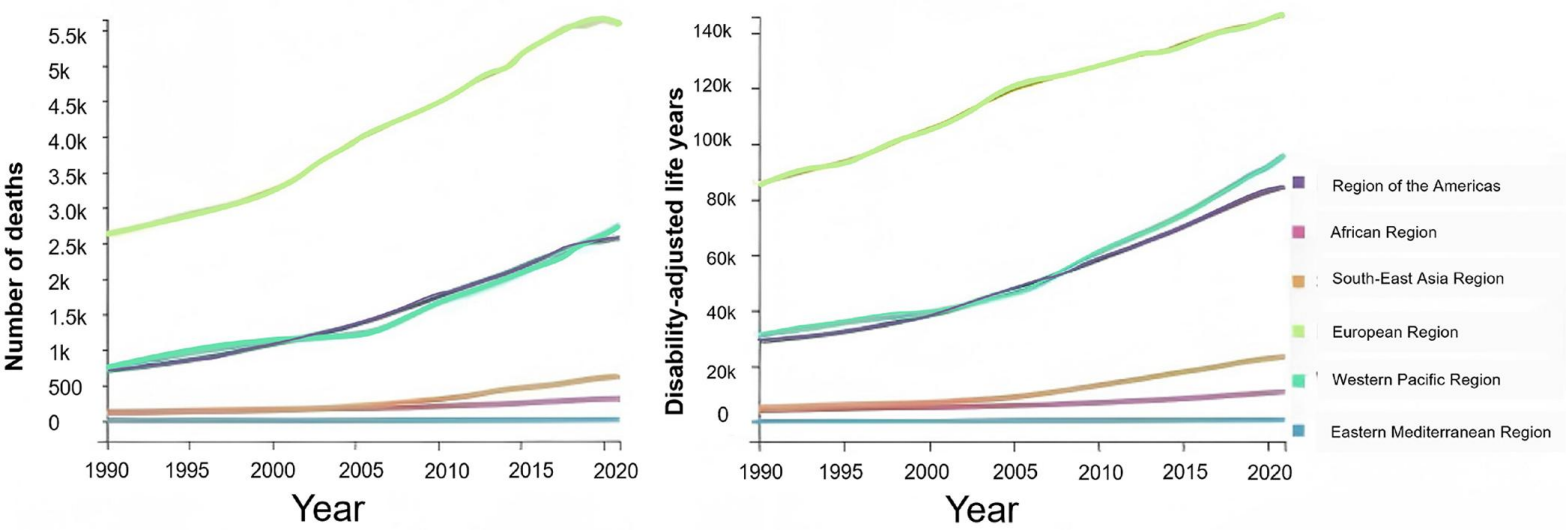
Alcohol-attributable AF-related mortality and disability-adjusted life years (DALYs) by wHO region, 1990-2021. Data source: GBD Study 2021. Case definition: ICD-10 I48.0-I48.9. Metric: Age-standardized alcohol-attributable mortality rate and age-standardized alcohol-attributable DALYs per 100,000 population. Alcohol-attributable fraction methodology: Based on population-level alcohol exposure, relative risks derived from systematic reviews, and the theoretical minimum risk exposure level. Model: CODEm for underlying mortality; GBD comparative risk assessment framework for alcohol-attributable burden estimation. Regions: Europe, the Americas, the Western Pacific, Southeast Asia, Africa, and the Eastern Mediterranean (WHO classification). Adjustment: Age- and sex-standardized; comorbidities (hypertension, diabetes, obesity) and healthcare access incorporated as covariates. Uncertainty: Shaded areas represent 95% UI. Design note: Figure has been split into two panels to improve readability.

According to WHO regional classifications, the impact of alcohol on AF-related mortality varies across different regions. Alcohol-related AF deaths are higher in Europe, with a slight stabilization in recent years. The Western Pacific and Americas regions have shown an increasing trend, while Africa, Southeast Asia, and the Eastern Mediterranean regions have relatively low and stable alcohol-related mortality. The trend in Disability-Adjusted Life Years (DALY) also follows a similar pattern (Figure 4).

### Gender and age-specific alcohol-related mortality trends

**Figure 5:**
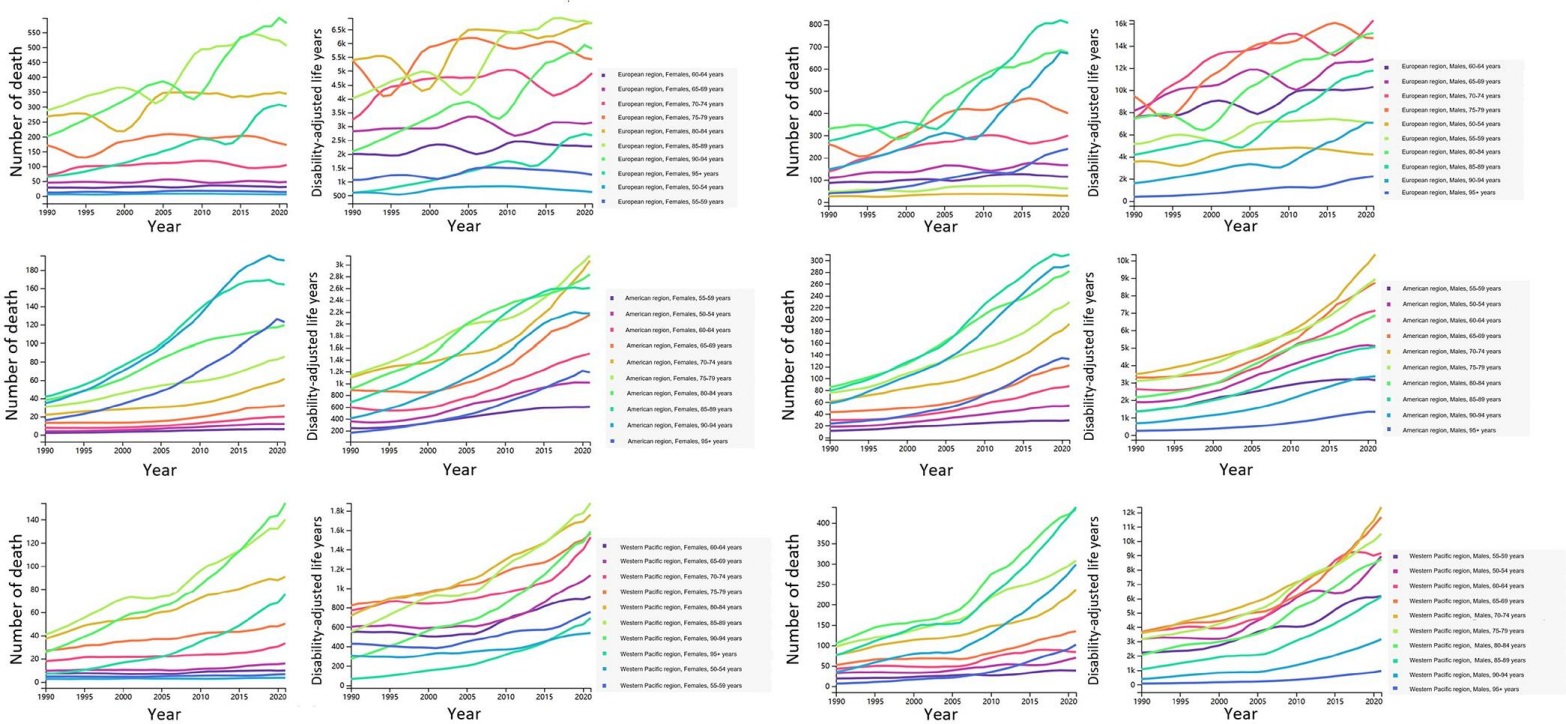
Age-specific alcohol-attributable AF mortality in selected WHO regions by sex, 1990-2021. Data source: GBD Study 2021. Case definition: ICD-10 I48.0-I48.9. Metric: Age-specific alcohol-attributable mortality rate per 100,000 population. Regions shown: Europe, the Americas, and the Western Pacific (selected to highlight major effects). Alcohol-attributable fraction: Estimated using GBD comparative risk assessment methods based on regional drinking patterns, alcohol use disorder prevalence, and AF-specific relative risks. Model: CODEm ensemble with integrated risk factor modeling. Adjustment: Age- and sex-specific; comorbidities and healthcare access covariates included. Uncertainty: Shaded ribbons indicate 95% UI. Design note: Figure simplified by sex-stratified panels and focused on regions with the highest alcohol-related AF burden.

In Europe, the mortality rate in women is higher in the 85–94 years group, particularly showing a significant increase in the 90–94 years group. In men, the highest mortality age groups are 80–94 years, with a significant increase observed in the 90–94 years group. In the Americas, the primary age group with the highest mortality is 85–94 years. In the Western Pacific region, the mortality rate in women aged 85–94 years has increased significantly, and in men, the mortality rate in the 80–89 years group has also risen (Figure 5).

### SDI regional differences in alcohol-related mortality

**Figure 6:**
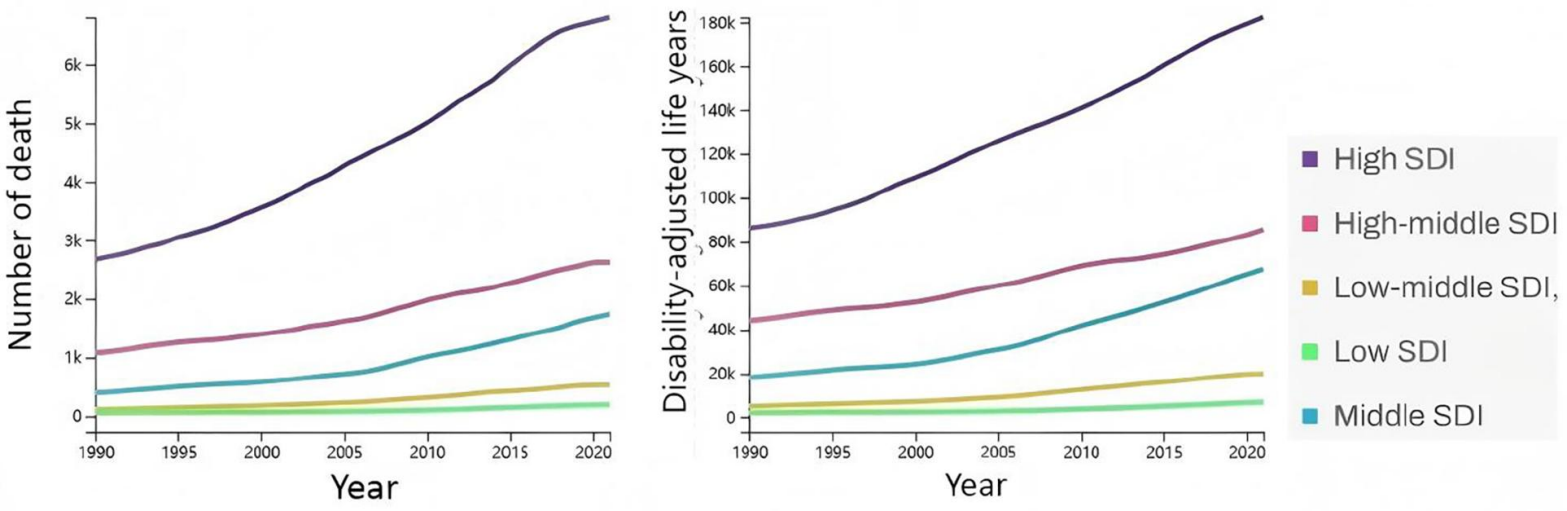
Socio-Demographic Index (SDI)-specific trends in alcohol-attributable AF mortality, 1990–2021. Data source: GBD Study 2021. Case definition: ICD-10 I48.0–I48.9. Metric: Age-standardized alcohol-attributable mortality rate per 100,000 population. SDI classification: Countries stratified into five quintiles (low, low-middle, middle, high-middle, and high SDI) based on income per capita, educational attainment, and fertility rate. Alcohol-attributable fraction: Population-level alcohol exposure and AF-specific relative risks applied within the GBD comparative risk assessment framework. Model: CODEm ensemble estimates. Adjustment: Age- and sex-standardized; comorbidities and healthcare access adjusted within GBD models. Uncertainty: Shaded areas represent 95% UI.

**Figure 7:**
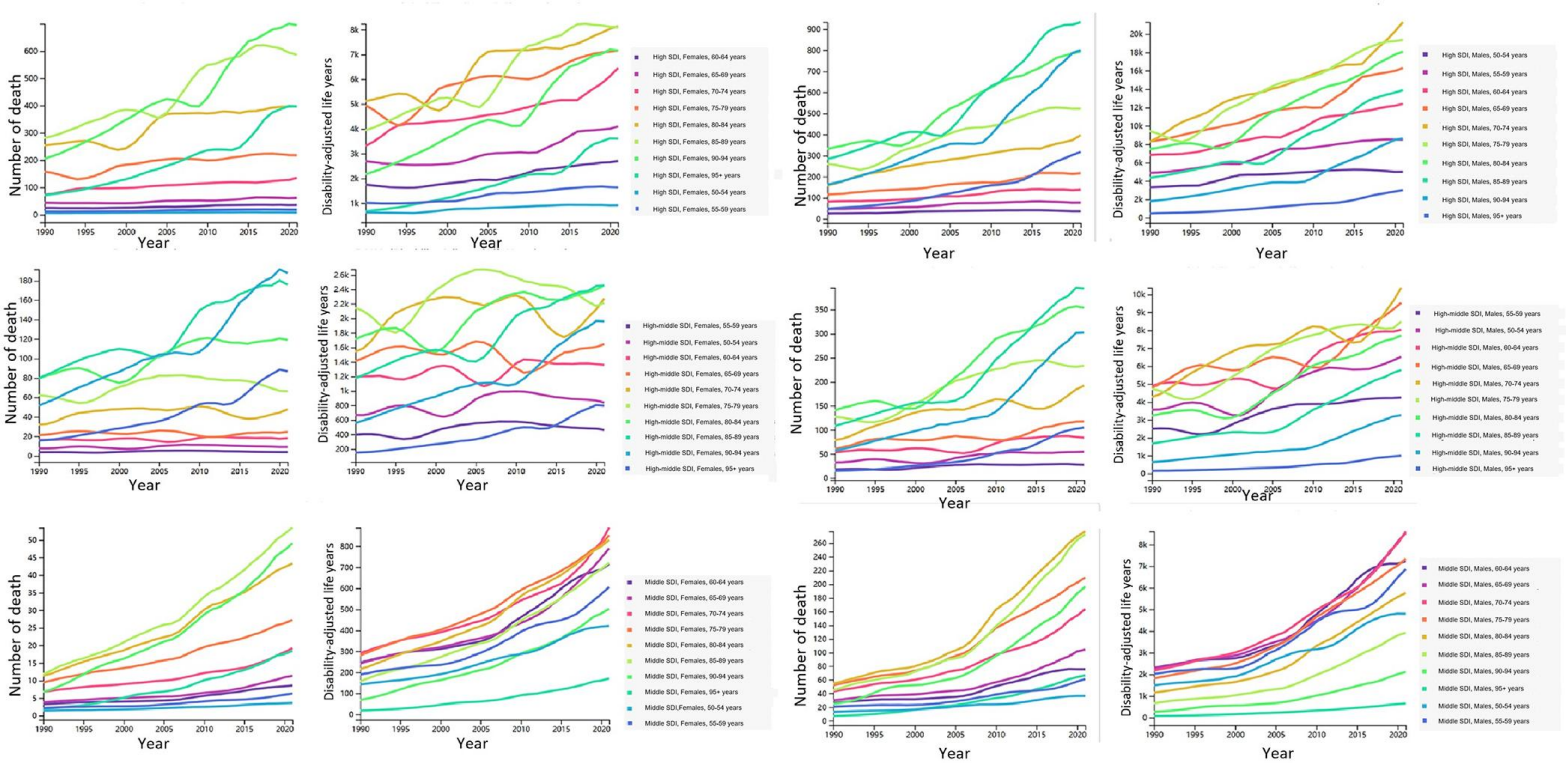
Age- and sex-specific alcohol-attributable AF mortality in high and high-middle SDI regions, 1990–2021. Data source: GBD Study 2021. Case definition: ICD-10 I48.0–I48.9. Metric: Age-specific alcohol-attributable mortality rate per 100,000 population. SDI groups: High SDI and high-middle SDI quintiles. Sex/age stratification: Data presented by sex (male/female) and age group (80–89, 85–94, 90–94, 95+ years), focusing on older adult populations where alcohol-attributable mortality is most pronounced. Alcohol-attributable fraction: GBD comparative risk assessment using regional alcohol exposure and AF-specific relative risks. Model: CODEm ensemble modeling. Adjustment: Age- and sex-specific estimates with comorbidity and healthcare access covariates. Uncertainty: Shaded ribbons denote 95% UI. Design note: Presented as a focused panel to highlight the major effect of alcohol in high-SDI settings; detailed multi-SDI age-stratified data are provided in Supplementary Figure X.

In high SDI regions, the alcohol-related AF deaths are generally higher, while low SDI and lower-middle SDI regions maintain lower and stable mortality rates (Figure 6). In high SDI regions, the mortality rate in women aged 85–94 years has increased, while in men, the mortality rate in the 80–94 years group has shown a significant increase. This trend is also noticeable in middle-high SDI regions (Figure 7).

### Sensitivity analysis

To ensure the robustness of our findings, we conducted sensitivity analyses. The results from these analyses were consistent with our primary findings, indicating that our conclusions remain valid across different assumptions and scenarios.

## Discussion

### Global trends in AF mortality

This study provides an in-depth analysis of the trends in AF-related mortality over the past three decades, based on the 2021 Global Burden of Disease (GBD) data. Our findings confirm the growing global burden of AF, with notable increases in AF-related deaths observed across both high-income and low-income regions. While previous studies have documented the rising incidence of AF globally, this study highlights significant regional disparities in mortality rates, suggesting that social, environmental, and healthcare-related factors play crucial roles in shaping AF outcomes.

Our results show that AF mortality has increased most markedly in middle- and high-income regions, particularly in Europe and the Western Pacific. This finding is consistent with previous research, which links the rising mortality in these regions to the aging population, increased awareness and diagnosis of AF, and improved survival from other cardiovascular diseases, such as myocardial infarction and heart failure, which may predispose individuals to AF (8). Moreover, high levels of comorbidities, such as hypertension, diabetes, and obesity, exacerbate the progression of AF and increase the risk of associated mortality (9). However, it is important to note that the increase in mortality is not uniform; some countries, including those in Scandinavia, have seen improvements in AF-related outcomes, potentially due to early detection, effective anticoagulation therapy, and the implementation of specialized AF management protocols (10).

In contrast, LMICs have also experienced an uptick in AF-related deaths, driven by improvements in life expectancy and rising prevalence of cardiovascular risk factors. In these regions, healthcare infrastructure may struggle to manage the growing burden of AF, leading to delays in diagnosis and inadequate treatment. As the risk factors for AF, including hypertension and metabolic disorders, continue to rise in these regions, the burden of AF is expected to grow further in the coming decades (1, 9).

### Impact of alcohol consumption on AF mortality

One of the most striking findings of this study is the significant impact of alcohol consumption on AF mortality, particularly in high-income regions such as Europe and North America. Previous research has consistently shown that alcohol use, especially chronic heavy drinking, is a major risk factor for AF, and this study reaffirms the association between alcohol-related AF deaths and the mortality trends observed in Europe (11). The Western Pacific region also exhibited a similar trend, likely due to the increasing consumption of alcohol in countries like Japan and South Korea, where AF-related mortality has also been rising (2).

The mechanism linking alcohol consumption and AF is well-documented, with alcohol-induced changes in atrial electrical properties, increased sympathetic tone, and structural remodeling of the heart being key contributors (6). Acute alcohol intake, particularly binge drinking, has been shown to precipitate AF episodes, while chronic alcohol abuse leads to atrial dilation and fibrosis, further increasing the risk of persistent AF (12, 13). Interestingly, the higher mortality rates associated with alcohol consumption in high-income regions may reflect both the direct effects of alcohol on the heart as well as the complex interactions with other risk factors, such as obesity, hypertension, and heart failure (9), which are more prevalent in these populations.

It is also important to consider tobacco smoking, which exhibits marked regional heterogeneity and frequently clusters with alcohol consumption as a shared lifestyle risk factor, particularly in regions such as Europe and the Western Pacific where both exposures are prevalent (14). Independent of alcohol, smoking promotes atrial fibrillation through systemic inflammation, oxidative stress, enhanced sympathetic activity, and direct atrial structural remodeling with interstitial fibrosis (6); moreover, combined smoking and heavy alcohol use appear to exert synergistic cardiovascular toxicity that substantially exceeds the additive risk of either exposure alone, likely through convergent profibrotic and proarrhythmic mechanisms (15). We acknowledge that the GBD 2021 provides population-level aggregated data without individual-level smoking status, precluding formal adjustment for tobacco use; consequently, our alcohol-attributable fractions may partially capture the joint effect of these correlated behaviors, and disentangling their independent contributions to AF mortality will require future individual-level cohort studies with granular lifestyle phenotyping.

Despite the well-established link between alcohol and AF, our study also highlights that alcohol-related mortality rates remain relatively stable or lower in low- and middle-income regions. This could be attributed to lower per capita alcohol consumption, as well as different drinking patterns, such as less frequent binge drinking in some LMICs (16). However, as these regions undergo economic development and the consumption of alcohol increases, a rise in alcohol-related AF deaths is likely to follow, underscoring the need for targeted public health interventions to mitigate the risks of alcohol consumption. Additionally, we acknowledge that physical inactivity and poor diet frequently cluster with alcohol consumption and may confound the observed associations, but the aggregated nature of GBD data precludes direct adjustment for these lifestyle factors.

### Sex- and age-specific mortality trends

Our study also explored sex- and age-specific trends in AF-related mortality, revealing distinct differences in mortality patterns. In women, AF-related deaths were concentrated in the older age groups (90–94 and 95+), while in men, the mortality increase was observed in both younger (30–44) and older (85+) age groups. This finding aligns with previous studies showing that AF-related deaths occur at younger ages in men compared to women, likely due to the earlier onset of cardiovascular diseases in men (17). Moreover, the higher mortality rates in older women may reflect the increased vulnerability of women to AF and its complications, as well as potential gender disparities in access to healthcare and treatment (18).

The observed age-related patterns also emphasize the need for age-tailored prevention and management strategies. Early detection and intervention in younger populations, particularly those with modifiable risk factors such as hypertension and obesity, could potentially reduce the burden of AF-related mortality in the long term (19). Similarly, for older adults, comprehensive management of comorbidities and a focus on preventing stroke and heart failure in those with AF may help mitigate the rising mortality rates observed in this age group (20).

### Socioeconomic and healthcare system factors

The findings of this study underscore the critical role of socioeconomic factors, particularly in high-income countries, where AF-related mortality rates are higher. Regions with higher SDI scores, such as those in Europe and North America, tend to have more advanced healthcare systems and greater awareness of AF (21). However, the rising rates of AF-related deaths in these regions point to the need for more effective public health strategies, particularly in addressing lifestyle factors such as alcohol consumption, obesity, and hypertension (22).

In LMICs, the increasing burden of AF is compounded by challenges in healthcare access and infrastructure (23). Early detection of AF is often delayed, and access to effective anticoagulation therapy, rhythm control strategies, and appropriate stroke prevention remains limited (24). As these regions continue to develop economically, strengthening healthcare systems and improving access to AF-related care will be essential to manage the growing burden of AF and reduce related mortality.

## Limitations

This study contributes to our understanding of the global burden of AF, but several important questions remain. Future research should aim to explore the underlying mechanisms of regional disparities in AF mortality, particularly in LMICs, where the burden is expected to grow rapidly. Additionally, the role of emerging risk factors, such as the impact of air pollution and climate change on cardiovascular health, warrants further investigation. Longitudinal studies examining the effectiveness of public health interventions, such as alcohol reduction programs and hypertension control initiatives, could provide valuable insights into how to mitigate the rising AF mortality in at-risk populations.

## Conclusion

In conclusion, our study highlights the increasing global burden of AF-related mortality, with significant regional, sex, and age differences. The impact of alcohol consumption remains a major contributor to AF-related deaths, particularly in high-income regions, while the rising prevalence of AF in LMICs presents a growing public health challenge. Efforts to improve early detection, reduce alcohol consumption, and address cardiovascular risk factors, particularly in high-risk populations, will be essential to reduce AF-related mortality in the future.

## Data Availability

The original contributions presented in the study are included in the article/Supplementary Material, further inquiries can be directed to the corresponding authors
